# Surface roughness and shear bond strength of novel polymethylmethacrylate-based materials

**DOI:** 10.1007/s10266-025-01138-w

**Published:** 2025-06-25

**Authors:** Nursena Öztemel, İrem Beril Yeşil Kurt, Almira Ada Diken Türksayar

**Affiliations:** Department of Prosthodontics, Faculty of Dentistry, Biruni University, Istanbul, Turkey

**Keywords:** Graphene, PMMA, Shear bond strength, Surface roughness, CAD-CAM

## Abstract

The aim was to evaluate the surface roughness (Ra) and shear bond strength (SBS) of novel polymethylmethacrylate (PMMA)-based materials. Thirty rectangular specimens were prepared from three different PMMA-based materials: nanographene-reinforced PMMA, milled PMMA, and self-curing PMMA. All specimens underwent a polishing process and the Ra value was then recorded using a non-contact profilometer. A special plate was then used to bond nanoflowable composite resin to the specimens' surfaces, after which they underwent thermocycling between 5 and 55 °C for 5000 cycles. The SBS was subsequently evaluated at a crosshead speed of 1 mm/min. One-way ANOVA and Tukey's HSD test (α = 0.05) were used for data analysis. The Ra and SBS values differed significantly (*p* < 0.001). The self-curing PMMA specimens had the highest mean Ra value (1.15 μm), while the G-CAM specimens had a mean Ra value of 0.55 μm and the milled PMMA specimens had a mean Ra value of 0.53 μm. SBS in the milled PMMA group was measured at 51.25 MPa; SBS in the G-CAM and self-curing PMMA specimens was lower than in the milled PMMA specimens (34.36 and 31.22 MPa, respectively). Although nanographene-reinforced PMMA exhibited an acceptable Ra value, its SBS value was lower than that of milled PMMA.

## Introduction

Interim prostheses can be produced through using either conventional or computer-aided design, and computer-aided manufacturing (CAD-CAM) technologies [1, 2]. Interim restorations are used to restore the tooth structure providing both biological and mechanical protection to the remaining tooth [3, 4] as well as restoring implants during and after the osteointegration process to maintaining esthetics and shape the surrounding soft tissue and restore proper occlusion and function [5, 6]. Polymethyl methacrylate (PMMA) is often chosen as it is easy to use and has a low elastic modulus, which helps prevent stress from occlusal forces [7]. Milling PMMA disks with CAD/CAM technology facilitates the fast and precise fabrication of provisional restorations [8]. While prepolymerised PMMA has a similar chemical makeup to conventional PMMA, processing it at high temperatures and pressures significantly improves its mechanical properties [9–11]. Besides the mechanical properties,, the bond strength of the material is essential for ensuring long-term clinical stability [12, 13]. The ability to easily modify and repair interim restorations is essential, as this characteristic significantly influences the overall success or failure of the treatment outcome [14]. This is particularly important when preparing of the correct emergence profile for implant-supported prostheses. Following implant surgery, resin-containing materials are used for chairside adjustments to the prepared restoration, in anticipation of potential soft tissue alterations that may occur during the surgical procedure [15]. These adjustments also serve to obliterate the buccal screw holes [16]. Nano-flowable composite resin was selected as the bonding material in this study due to its common clinical applications, such as repairing fractured restoration and adjusting the emergence profile, particularly in implant-supported and provisional restorations. Its low viscosity and ability to adapt to fine surface details make it especially suitable for these purposes. Accordingly, its incorporation into the SBS test demonstrates its clinical relevance and practical application in the correction or enhancement of 3D-printed restorations [17, 18].

The development of nanotechnology has enabled graphene to be used as a reinforcing agent in polymers such as PMMA, representing a significant advance in the subtractive manufacturing of restorative materials [19–21]. Graphene is a nanomaterial characterized by a two-dimensional honeycomb structure, with carbon atoms integrated into its main framework [22, 23]. There are various forms of graphene, including graphene sheets, graphene oxide (GO) and reduced graphene oxide (rGO) [24–26]. As a relatively novel material, graphene oxide has been shown to exhibit several promising characteristics that make it a potentially valuable addition to prosthodontics. These characteristics include biocompatibility, biodegradability, a high Young's modulus, transparency, flexibility and reduced microbial adhesion. [27, 28]. Nanographene-reinforced PMMA has been introduced to the market for a range of applications, including veneers, inlays, onlays, crowns (supported by teeth or implants), as well as three-unit tooth-supported fixed partial dentures and complete dentures [29].

A number of studies have demonstrated that incorporating graphene into PMMA materials can enhance the resin’s mechanical properties and decrease shrinkage during the polymerization [30–32]. To evaluate the effects of this promising technology, previous studies have investigated the flexural strength, hardness, and color stability of graphene-reinforced PMMA [7, 33–36]. Nevertheless, there is a paucity of data regarding the shear bond strength of graphene-reinforced PMMA materials in the literature. Therefore, the objective of the present study is to investigate the surface roughness (Ra) and shear bond strength (SBS) of three different PMMA materials (nanographene-reinforced PMMA, milled PMMA, self-curing PMMA) bonded to composite resin. The first null hypothesis was that the material type would not affect the Ra (I) and the second null hypothesis was that the material type would not affect the SBS to the nanoflowable composite resin (II).

## Materials and methods

**Fig. 1:**
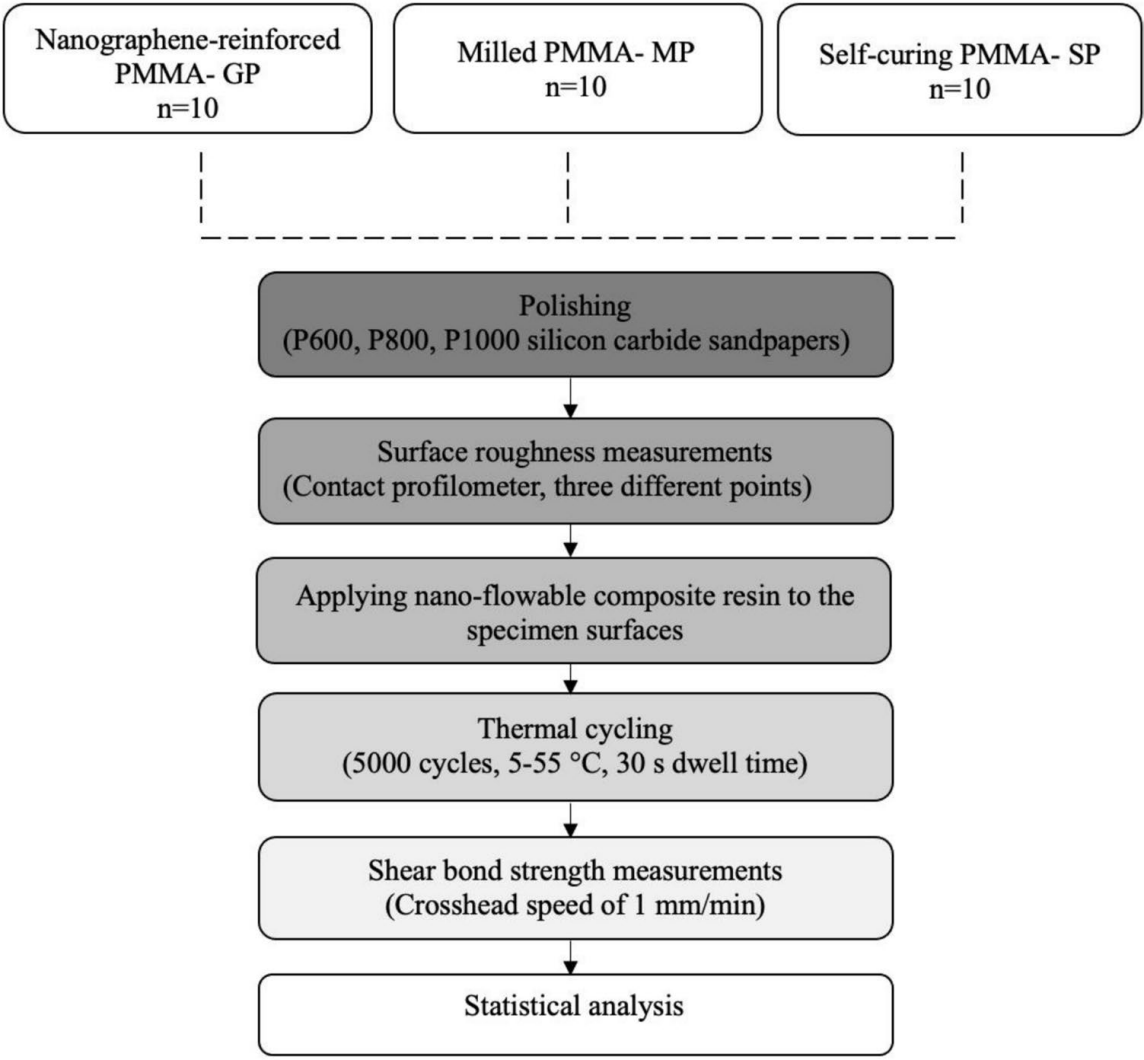
Overview of the study

The number of specimens required for the study was calculated based on a previous study evaluating the mechanical properties of the G-PMMA material [37]. Power analysis (G*Power 3.1 from Heinrich Heine University) was used to determine the total specimen size and it was determined as six per group (*f* = 1.1, 1 − α = 0.05, 1 − β = 0.95). However, to enhance the statistical power of the study, ensure the reliability of the results, and fortify the findings, the number of samples for each group was increased to 10. The studies were conducted for the shear bond strength test using rectangular specimens measuring 15 × 15x3 mm. Three study groups were examined, using two different milled PMMA resin materials [nanographene-reinforced PMMA (G-CAM, Graphenano Dental, Graphenano Nanotechnologies, Spain; GP), milled PMMA (Tempo CAD, On Dent; MP)] and one self-curing PMMA (Imident, Imicryl; SP) (*n* = 10). Figure 1 shows the overview of the present study.

In the GP and MP groups, rectangular specimens were prepared using a precision cutting machine (Micracut 151, Metkon, Bursa, Turkey) and CAD/CAM disks. For the SP group, specimens with equivalent dimensions to those in other groups were fabricated using silicone molds in accordance with the manufacturer's guidelines. The dimensions of the specimens in all groups were subsequently checked using a digital caliper. The rectangular specimens were underwent wet grinding with P600, P800, and P1000 silicon carbide sandpaper under water for five minutes, and were then subjected to ultrasonic cleaning in distilled water. A contact profilometer (Marsurf PS1; Mahr) was used to measure Ra values. The measurements were obtained at three different points on the specimens (stylus tip radius: 5 µm, stylus driving speed: 0.5 mm/s, traversing length: 1.75 mm, and five cut-off lengths: 0.25 mm). The Ra value for each specimen was determined by calculating the average values of these measurements.

For the SBS test, the specimens were treated with Single Bond Universal (3 M ESPE) and after which they were polymerized using a light-emitting diode (LED) unit (Bluephase; Ivoclar Vivadent AG, Schaan, Principality of Liechtenstein) at a light intensity of 1200 mW/cm^2^ for 20 s. A custom metal plate was then fabricated to ensure the composites could be applied consistently. Nano-flowable composite resin in shade A2 (Dline, Siauliai, Lithuania) was then applied to the specimen surfaces using a special metal plate, followed by light-emitting diode polymerization for 30 s.

The specimens underwent thermal aging in a thermocycler (5000 cycles, 5–55 °C, dwell time of 30 s). They were then subjected to a shear bond strength test, during which they were affixed to a predesigned metal plate and evaluated using a universal testing machine at a crosshead speed of 1 mm/min. (MIC-101, MODDENTAL, Turkey). As a result of the test, the data obtained were recorded.

The SBS value was calculated via the following formula:

Force to failure/bonding area; MPa = N/mm2. r = F/A formula, where is the SBS (MPa), F is the fracture load (N), and A is the bond area (mm^2^) [38].

An stereomicroscope with a magnification of x40 (PST 901, Metkon, Turkey) was used to visually inspect the fractured surfaces and assess the failure modes of the specimens. The failure modes were then classified as follows [39]:

Group A: adhesive failure (no nanoflowable composite remnants on the polished specimen surface.

Group C: cohesive failure (failure within the specimens or composite resins).

Group AC: combined adhesive failure and cohesive failure (exposure of luting cement remnants and polished specimen surface).

Following the SBS test, representative specimens were examined using a scanning electron microscope (SEM) at an acceleration voltage of 10.0 kV. The measurements were performed under 500, 1000, and 2000 magnifications and images were recorded. The descriptive statistics were calculated for statistical analysis using SPSS V23 (SPSS, Inc., Chicago, IL), and the Shapiro–Wilk test was used to assess data distribution. The data obtained were subsequently analyzed using one-way ANOVA, followed by Tukey's HSD test (α = 0.05).

## Results

A statistically significant difference was observed in the Ra values of the groups (*p* < 0.001). The mean values of Ra, standard deviation and median Ra values for the specimens are displayed in Table 1. The SP specimens exhibited the highest mean surface roughness (*p* < 0.001). GP and MP specimens had lower mean surface roughness (*p* = 0.94). All Ra values exceeded the clinically acceptable threshold of 0.2 μm.

**Table 1:** Descriptive statistics of Ra values of each material

Material	Mean ± SD	95% Confidence ınterval Lower- upper bound
MP	0.53 ± 0.09	0.46 – 0.59
GP	0.55 ± 0.03	0.53 – 0.57
SP	1.15 ± 0.30	0.93 – 1.36

Regarding the SBS values, a statistically significant difference was observed between the groups tested (*p* < 0.001) and these values, along with their standard deviations and median values, are presented in Table 2. The statistical analysis revealed that the MP group achieved significantly higher SBS values compared to the GP and SP groups (*p* < 0.001), while no significant difference was found between the GP and SP groups (*p* = 0.65) When failure analyses were evaluated, all specimens showed adhesive failure, regardless of fabrication method, except for one combined adhesive failure and cohesive failure in the MP group.

**Table 2:** Descriptive statistics of SBS values of each material

Material	Mean ± SD	95% Confidence ınterval Lower- upper bound
MP	51.35 ± 8.19	45.48 – 57.21
GP	34.36 ± 9.20	27.77 – 40.94
SP	31.22 ± 6.11	26.84 – 35.59

**Fig. 2:**
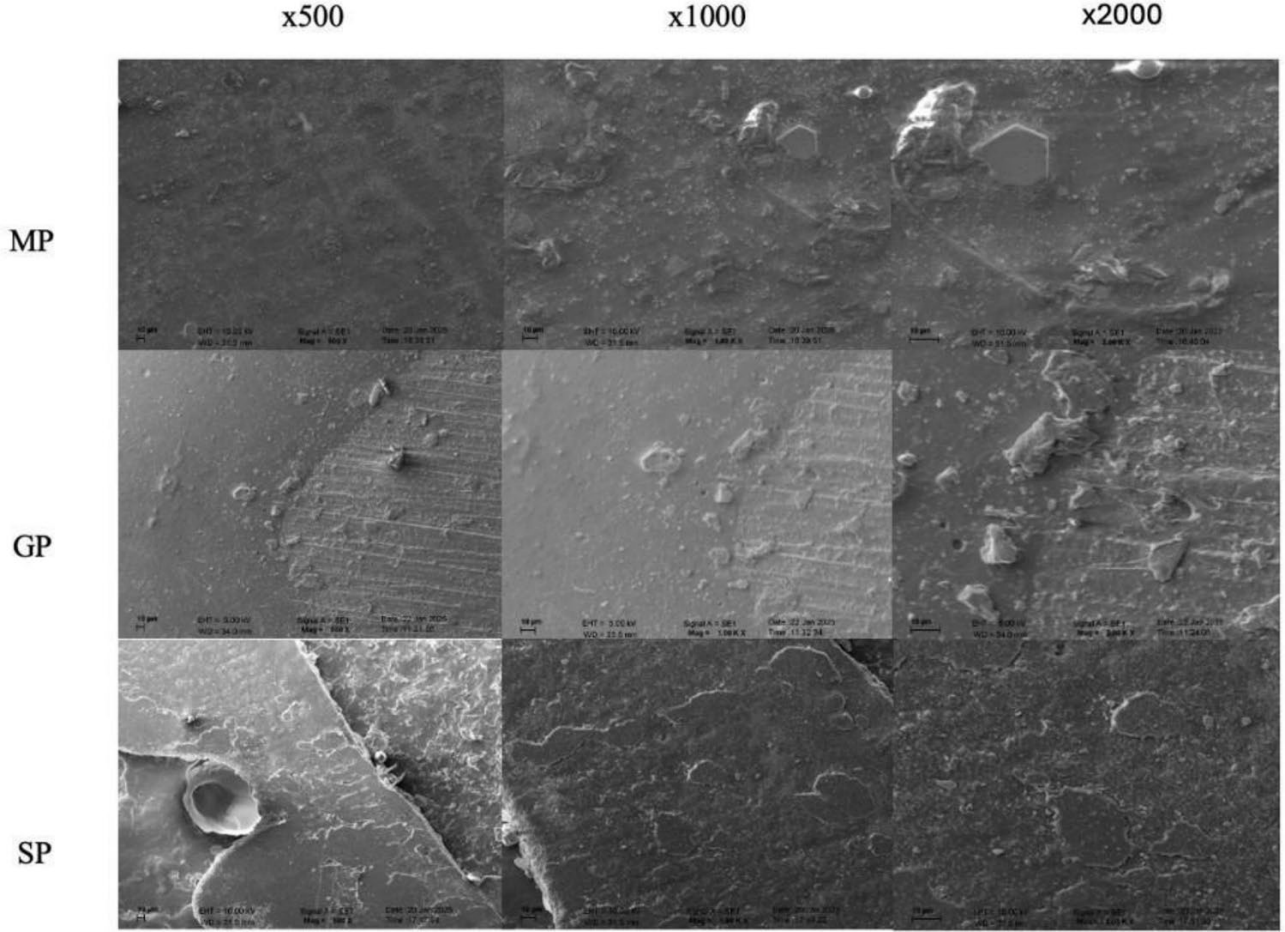
Examination of the surface images of the specimens with SEM Analysis. First column × 500, second column × 1000, and third column × 2000 magnification.

The images obtained from the SEM analysis showed similar results to the Ra values (Fig. 2).MP specimens exhibited a relatively smooth and homogeneous surface structure; however, minimal mechanical processing marks were observed due to the fabrication process. GP specimens exhibited pronounced scratches and heterogeneous particle distribution of particles on the surface, indicating that graphene doping had altered the surface properties. SP samples, on the other hand, had an irregular morphology with pronounced porosity and crack formation, which could be attributed to the effect of the autopolymerization process on the mechanical and structural integrity of the material.

## Discussion

The first null hypothesis, which stated that material type would not influence Ra, was rejected as the material type was found to have a significantly affect Ra. Although none of the specimens exhibited Ra values at or below the clinically acceptable threshold of 0.2 μm [40], significant differences in Ra values were observed among the materials. Previous studies have shown that conventional PMMA resins have higher Ra values than milled PMMA resins [10, 41]. Likewise, in the present study, self-curing PMMA specimens showed highest Ra values. MP and GP showed similar mean Ra values. The incorporation of nanoparticles into the PMMA did not result in a significant change to the Ra value of the material, which is consistent with a previous study on G-PMMA and milled PMMA [28]. One study assessed the influence of coffee thermocycling on the Ra of nanographene-reinforced PMMA compared to prepolymerised PMMA, finding that the mean Ra values were similar following polishing [11].

Surface roughness decreases biocompatibility and increases the risk of surface fatigue [20]. Increased Ra can induce biofilm formation, which may have a negative impact on gingival health and the compatibility of soft tissues with the restoration [2, 6]. In this study, the higher Ra values of self-curing PMMA specimens may be attributed to the powder-to-liquid ratio and the conventional manufacturing process. Porosity, resin shrinkage, and residual monomer can all negatively affect the chemical, physical, and mechanical properties of resin-based restorations produced with conventional manufacturing methods [26].

The second null hypothesis was rejected as well, as milled PMMA exhibited the highest mean SBS value of all the groups. All specimens had an SBS value above the acceptable threshold of 5 MPa, as specified by the International Organization for Standardization (ISO) 10,477–2020 standard [42]. Moreover, the SBS values of all the specimens were above the minimum clinical SBS value of 10 MPa reported in the literature [43]. However, incorporating nanoparticles into the PMMA structure did not result in a significant change in its bond strength. Almost all specimens exhibited adhesive failure. These findings were aligned with the SBS values of all specimens. Although the SBS values are higher than the acceptable threshold, the presence of adhesive failure indicated the need for additional surface treatments. A previous study assessed the SBS of CAD/CAM and conventional interim materials and concluded that CAD/CAM interim materials exhibit enhanced mechanical properties, making them more suitable for clinical applications than conventional materials [13]. The bond strength following the repair procedure is primarily determined by the extent of unconverted C = C double bonds [44]. As polymerization progresses over time, the concentration of C = C double bonds decreases, while the extent of polymer cross-linking increases [4]. Since the milled PMMA specimens used in this study were prefabricated and the conventional specimens were evaluated shortly after production, it is expected that the self-curing specimens will contain a higher concentration of unconverted C = C double bonds [13]. The authors are not aware of any prior studies examining the SBS of nanographene-reinforced PMMA in bonding to composite resin, making it impossible to compare the results with existing research on these materials. Nevertheless, a recent study investigated the SBS of G-PMMA with self-adhesive resin cement to various implant abutment materials and additive-manufactured resin materials. It was reported that milled nanographene-reinforced PMMA mostly exhibited the lowest bond strength values among the tested materials. However, this result should be interpreted with caution since the study was conducted with different materials [21].

The intraoral application of resin composite is a less time-consuming and cost-effective method of restoring fractures and implant-supported prostheses in terms of shaping the surrounding soft tissues. Furthermore, this method eliminates the necessity for reproduction of the restoration and reduces the time spent at the chairside.

While this study is original in evaluating the SBS of G-CAM material, it has certain limitations. Using a single nanoflowable composite resin may have affected these results. A further limitation of the study is that nanographene-reinforced PMMA was only compared with subtractively and conventionally manufactured materials. Future studies should include testing with additively manufactured materials and different surface treatments to improve bonding success. Furthermore, the findings should be supported by additional bond strength tests, including tensile bond strength tests.

## Conclusions

Based on the results of this in vitro study, it can be concluded that:

1. After polishing, none of the evaluated materials achieved an Ra value within the clinically acceptable threshold.
2. GP showed acceptable Ra values but lower shear bond strength compared to MP.
3. All specimens demonstrated adhesive failure despite the SBS values being higher than the acceptable threshold.

## Data Availability

All data included in this study are available upon request by contact with the corresponding author.
